# Comparative dosimetric evaluation of 68Ga-PSMA and 18F-Choline PET/CT imaging in prostate cancer: implications for radiation safety and SUVmax correlation

**DOI:** 10.3389/fnume.2025.1702390

**Published:** 2025-12-01

**Authors:** Hussein R. Kaafarani, Mohamad Haidar, Hanna El-Balaa

**Affiliations:** 1 Department of Nuclear Medicine, American University of Beirut Medical Center, Beirut, Lebanon; 2 Department of Nuclear Medicine, American University of Beirut (AUB), Beirut, Lebanon; 3 Medical Physics (Beirut/Hadath), Lebanese University, Beirut, Lebanon

**Keywords:** PET/CT, dosimetry, ^68Ga-PSMA, ^18F-Choline, prostate cancer, radiation safety, SUVmax

## Abstract

**Background:**

Comparative patient dosimetry for diagnostic PET/CT can guide radiation-safety procedures and tracer selection in prostate cancer. We compared [^68Ga]Ga-PSMA-11 and ^18F-choline PET/CT and examined whether kidney SUVmax predicts patient effective dose (ED).

**Methods:**

Prospective single-center study of consecutive men undergoing clinically indicated PET/CT: 70 ^68Ga-PSMA-11 and 70 ^18F-choline examinations (Jan 2022–Dec 2023). Dose-rate measurements were recorded at the injection site and at 1 m, immediately post-injection and at 1 h. ED was derived from literature-based tracer coefficients (h_PSMA-11 = 0.0169 mSv/MBq; h_F-choline = 0.0173 mSv/MBq). Kidney SUVmax was extracted in a subset (*n* = 40 per tracer) to test ED–SUVmax associations (Pearson's *r*).

**Results:**

Mean surface dose rate was higher with ^68Ga-PSMA vs. ^18F-choline (4.9 ± 0.8 vs. 4.5 ± 0.7 µSv·h^−^^1^; *p* = 0.004). At 1 m, the difference persisted but was smaller (1.9 ± 0.3 vs. 1.7 ± 0.3 µSv·h^−^^1^; *p* = 0.02). Effective dose (ED) was similar between tracers (21.3 ± 3.6 vs. 20.7 ± 3.4 mSv; *p* = 0.28). SUVmax correlated with ED for ^68Ga-PSMA (*r* = 0.71; *p* < 0.001), but not for ^18F-choline (*r* = –0.12; *p* = 0.46).

**Conclusions:**

^68Ga-PSMA yields slightly higher dose-rate readings than ^18F-choline, while overall ED is comparable. These exploratory correlations do not support SUVmax as a stand-alone safety surrogate or outcome predictor.

## 1 Introduction

The imaging of prostate cancer has developed and is further improving due to advancement of tracer chemistry and scanners. Recurrent disease comparative studies indicate that there can be a difference in the performance between PSMA ligands–such as head-to-head comparisons between [18F]PSMA-1007 vs. [68Ga]Ga-PSMA-11 demonstrating significant nuances in the lesion-identifying and lesion-staging pathways (1). The selection of the tracer also varies in accordance with the clinical question: in the case of the assessment of the presence of the osseous disease, PSMA PET/CT and skeletal agents that include the use of ^18F-NaF can present varying strengths and trade-offs (2). In addition to test accuracy, it has been theorized that tracer choice may have some cost-effectiveness consequences on pathways, and disparities between [^68Ga]Ga-PSMA-11 and [18F]PSMA-1007 have been reported in recurrent settings (3). The results of earlier hybrid-imaging-performing tri-modality PET/CT-MRI studies contrasting the use of 68Ga-PSMA and 11C-choline indicate that the biology of the acquisition platform and the biology of tracer converge to shape the detection (4). Other targets (e.g., bombesin antagonists) compared to -18F-choline and multiparametric MRI go further to point out the effects of biological targeting on primary-tumor conspicuity (5).

Measures of uptake are quantitative and offer clinical information. An elevated preoperative Sulphur-Uranus supranatal maximum in PSMA PET/CT correlate with considerably reduced biochemical-recurrence risk following prostatectomy in live ligands as [68Ga]Ga-PSMA-11 and [18F]DCFPyL (6), and several cohorts correlate PSMA-PET SUVs to poor biologic results (7). Imaging biomarkers also communicate with serologic markers in biochemical recurrence: correlational studies of PSA with SUVmax and total metabolic tumor volume on a tracer, [18F]PSMA-1007 and [18F-choline], show the relationship between tracer-specific uptake and disease burden (8). These differences in performance are synthesized in meta-analytic comparisons of the use of tries of [1]18F-labeled PSMA agent and [68Ga]Ga-PSMA-11 in the clinical setting in a meta-analytic manner (9). Methodologically, there has been an increased interest in whether simple image measures could be taken to be dose relevant [i.e., could there be a correlation between SUVmax on [68Ga]/Ga-PSMA ligands or [18F]choline and patient radiation dose (10). Simultaneously the clinical variability can be observed: the selected cases show that [^18F]fluoromethylcholine can identify recurrence in case of [^68Ga]Ga-PSMA-11 unchecked (11), and that the wider staging/restaging comparisons between [^68Ga-PSMA] and [18F]choline define complementary activities at different phases of the disease (12). More recent prospective studies using other PSMA chemistries (e.g., (18F)ALF-PSMA-HBED-CC as compared to 68Ga-PSMA-HBED-CC) can be used to expand those contrasts to intermediate/high-risk staging (13).

Protocol and hardware developments are also important. Count statistics, noise properties and practicability of dose versus image quality can be varied with long axial field-of-view with ultra-high sensitivity (14). Multimodality considerations have also been demonstrated using case-based reports where the interpretation of both the 18F-PSMA-1007 and the 18F-FDG were used in advanced disease (15). Safety-wise, it is possible to construct local diagnostic reference levels of whole-body PET/CT and anchor the protocol optimization, as well as comparative dosimetry across the procedures of 18F-choline and 68Ga-PSMA so that the exposure of the patients can be quantified in routine practice (16) and (17). The PSMA toolkit is further expanded with tracer development, such as the use of [^18F]PSMA-1007 to detect local relapse in specific situations (18) and network meta-analyses to combine the evidence of modalities and prostate cancer treatment steps (19). Physiological and off-target patterns should also be taken into consideration during interpretation; heterogeneous PSMA, choline, and FDG uptake in ganglia is the harmonic example of the pitfalls that should be avoided in the context of specificity and reader training (20).

The dosimetric model used in this study is based on the conventional standards. The concepts of effective dose and principles of protection are based on the ICRP recommendations (21), and the dose coefficients of most commonly used substances have been collected (22). In converting DLP to CT-related effective dose, conversion factors depending on the area of the whole population being scanned must be used (23). Specific labeling ([68Ga]Ga-PSMA-11) provide information on activity levels administered and side effects data utilized in clinical procedures (24). The dosimetry nomenclature and schema follow MIRD conventions (25), and domestic estimates of internal dose can be supplemented with OLINDA/EXM where available (26). E-PSMA standardized reporting guidance (27) and the joint EANM/SNMMI procedure guideline of PSMA PET/CT 2.0 (28) are aligned with reporting and acquisition, and quantitative interpretation is conscious of partial-volume effects which may be used to bias small-lesion SUV values (29).

In this context, we analyze radiation exposure in [68Ga] Ga-PSMA-11 vs 18F-choline PET/CT and SUVmax as a secondary biomarker in order to inform the protocol decision-making process to balance diagnostic yield, safety and standardized reporting.

## 2 Materials and methods

### 2.1 Study design and setting

We conducted a prospective, single-center comparative study at the American University of Beirut Medical Center (AUBMC) from January 2022 to December 2023. The aim was to compare patient radiation metrics for ^68Ga-PSMA and ^18F-Choline during routine diagnostic PET/CT in men with prostate cancer. The study protocol was approved by the AUBMC Institutional Review Board (IRB #IRB20220894); Written informed consent was obtained in accordance with IRB policy. Having two times made it simple to compare the properties of physical dose exposure and tracer behavior, mostly in terms of renal uptake. The research team operated under the rules of the institution, and participants gave their informed consent. The two tracers could be compared, and it was found that there were differences in the radiation they produce and how they distribute in the body in PET imaging for cancers. Because of our decision to conduct the study at AUBMC, we were provided with sophisticated PET/CT imaging and professional experts to help with ensuring excellent standards of collecting and analyzing the data. Using this planning approach, the results of different types of tests could be accurately compared, and the protocol for nuclear medicine could be improved.

### 2.2 Patient cohort

We analyzed consecutive adult male patients referred for clinically indicated PET/CT for prostate cancer. Two tracer cohorts were included: ^68Ga-PSMA (*N* = 70) and ^18F-Choline (*N* = 70). Inclusion required complete PET/CT acquisition and scanner logs; exclusions were duplicate exams and technically compromised studies. A predefined SUVmax subset (*n* = 40 per tracer) was used for renal uptake analysis. We analyzed consecutive adult male patients referred for clinically indicated PET/CT for prostate cancer. Two tracer cohorts were included: ^68Ga-PSMA (*N* = 70) and ^18F-Choline (*N* = 70). Inclusion required a completed PET/CT examination with available scanner logs; exclusions were duplicate exams and technically compromised studies. A predefined subset (*n* = 40 per tracer) was used for renal SUVmax analysis. Patient age ranged from 34 to 64 years (mean 49.7 ± 10.2 years) and body weight from 60 to 100 kg (mean 77.2 ± 12.6 kg). Administered activity ranged from 185 to 370 MBq per institutional protocol. All procedures followed institutional safety and ethics standards.

### 2.3 Pet/Ct and radiotracer details

At AUBMC, PET/CT scans of the whole body were performed by following an established imaging protocol. Both ^68Ga-PSMA-11 and [^18F] F-choline were injected intravenously in accordance with the rules established at the institution. The amount chosen for ^68Ga-PSMA-11 was between 2.09 and 5.80 mCi, and for ^18F-choline ranged from 2.9 to 8.87 mCi, applied according to patient weight and set dosimetry guidelines. The researchers started imaging one hour after the injection, which matched the best spots where both tracers had reached in the body. PET/CT images were acquired on a [Biograph mCT] [(Siemens Healthineers), Erlangen, Germany], a time-of-flight–capable PET/CT system; acquisition and reconstruction followed the clinical protocol described below. They were made to lie flat on their backs, making sure their bodies were positioned carefully to avoid extra movements and get excellent images. Appropriate safety steps were applied, like having radiation shields, calibrating the syringe doses, and recording the time following the injection. Image collection was done by moving the patient from one axial bed position to another, covering the abdomen, pelvis, and thorax. All scans followed a specific uptake timeline and collected data for the same length of time to keep the results from varying. These simplified actions helped assess the activities of various radiotracers within patients and cut down on possible errors caused by different methods. PET/CT data were stored in the DICOM format and processed on approved workstations to measure SUV for quantitative purposes and to determine the right dose.

The scanner-reported DLP (mGy·cm) for the localization CT was converted to ED_CT (mSv) using ED_CT = DLP × *k*, with *k* = 0.015 mSv·mGy^−^^1^·cm^−^^1^ for adult abdomen–pelvis. ED_PET (mSv) = A_admin (MBq) × h_tracer (mSv/MBq) with h_PSMA-11 = 0.0169 mSv/MBq and h_F-choline = 0.0173 mSv/MBq. ED_total = ED_PET + ED_CT.

Whole-body PET/CT was performed on a single time-of-flight–capable PET/CT system [(Biograph mCT), (Siemens Healthineers), Erlangen, Germany] using a uniform clinical protocol. Low-dose CT for attenuation correction and anatomical localization used 120 kVp, automatic tube-current modulation (reference 30–50 mAs), pitch ∼1.0–1.2, collimation 64 × 0.6 mm, and rotation time 0.5 s; images were reconstructed with the vendor's standard iterative kernel. No IV contrast was administered. PET emission data were acquired from thorax–abdomen–pelvis with 2.0–3.0 min per bed position (vendor-default bed overlap). PET images were reconstructed with OSEM (3 iterations×21 subsets) with TOF and PSF enabled, matrix 256 × 256, and Gaussian 5–6 mm FWHM post-filter. Standard vendor corrections (attenuation, scatter, randoms, decay, dead-time, normalization) were applied. SUVs were normalized to body weight. Volumes of interest (VOIs) for renal cortex SUVmax were drawn on fused PET/CT while excluding adjacent bowel and hepatic activity; partial-volume considerations followed prior guidance cited in the manuscript (29).

### 2.4 Dose rate measurements

The dose rate of radiation was measured at two spots: on the skin of the patient (around the abdomen) and 1 meter from the patient. Scans for measurements were done right after tracer injection and again exactly an hour after administration. They were chosen to represent the drug's release and when its distribution in the body begins to drop. The levels of radiation detected in the samples were decreased by the background radiation value of 0.018 μSv/h, measured in a different space nearby. Dose-rate values were recorded but are reported and interpreted in the Results section. Within an hour, the activity from both tracers had dropped by a lot, indicating that the substance was being removed from the body as it should. They were key in measuring how much the workers were exposed and the type of emissions each tracer produced. All medical interventions adhered to the requirements established by the ICRP and the organization's radiation protection rules to ensure everyone's safety.

### 2.5 SUV-max measurement for kidneys

Values for kidney SUVmax were obtained on fused PET/CT at ∼60 min post-injection. A standardized VOI was drawn over the renal cortex on the fused images using certified analysis software; care was taken to exclude adjacent liver and bowel activity. SUV was computed as [activity concentration (kBq/mL)]/[injected activity [kBq]/body weight [g]]. Background signal was handled consistently across cases, and measurements used the same uptake time window to limit variability. In the predefined subset (*n* = 40 per tracer), mean kidney SUVmax was 38.4 ± 3.7 for ^68Ga-PSMA and 9.6 ± 1.2 for ^18F-Choline (range: 32.5–44.1 and 8.0–11.1, respectively). Methodological considerations regarding partial-volume effects and VOI definition are addressed in the Discussion and with supportive references.

### 2.6 Radiation dose calculations (ED) and correlation endpoint

For the abdomen–pelvis CT acquisition, the dose–length product (DLP, mGy·cm) reported on the scanner dose report was converted to effective dose (ED_CT, mSv) using ED_CT = DLP × *k*, with *k* = 0.015 mSv·mGy^−^^1^·cm^−^^1^ for adult abdomen–pelvis. This *k* = 0.015 mSv·mGy^−^^1^·cm^−^^1^ (adult abdomen–pelvis) is consistent with published adult conversion coefficients and AAPM guidance (21, 23, 24).

ED_PET (mSv) was computed as A_admin (MBq) × h_tracer (mSv/MBq) using tracer-specific, literature-based effective dose coefficients (27; 22): h_PSMA-11 (⁶⁸Ga) = 0.0169 mSv/MBq (U.S. FDA label for Gallium Ga 68 PSMA-11) and h_F-choline (^1^⁸F) = 0.0173 mSv/MBq (5.2 mSv per 300 MBq).

ED_total (mSv) = ED_PET+ED_CT. We report ED as mean ± SD by tracer and use two-sided tests (*α* = 0.05).

The prespecified primary analysis correlated kidney SUVmax with ED_total (mSv). Exploratory correlations involving ambient dose-rate (µSv/h) at fixed geometry/time were analyzed separately and are reported only in the Supplementary Material to avoid endpoint mixing.

### 2.7 Statistical analysis

Continuous variables are summarized as mean ± SD [or median (IQR) if skewed]. Normality was assessed with the Shapiro–Wilk test. Correlations between kidney SUVmax and ED *total* (mSv) were computed using Pearson's r when approximately normal; otherwise, Spearman's *ρ*. Where appropriate, variables were log-transformed; 95% CIs for r were derived using Fisher's z transformation. All tests were two-sided with *α* = 0.05. Analyses were performed in IBM SPSS Statistics, Version 26.0. The statistical analysis plan and correlation methods were reviewed by an independent biostatistician for appropriateness and clarity. In a secondary analysis, we correlated body mass (kg) with dose-rate (µSv/h) at ∼1 h post-injection for each tracer using Pearson's r (or Spearman's *ρ* if non-normal).

## 3 Results

We studied 140 consecutive men with prostate cancer undergoing diagnostic PET/CT (^68Ga-PSMA, *n* = 70; ^18F-choline, *n* = 70). Mean age was 49.7 ± 10.2 years (range 34–64) and mean body weight 77.2 ± 12.6 kg (range 60–100). Administered activity followed protocol (185–370 MBq), with imaging at ∼60 min post-injection; a predefined subset (*n* = 40 per tracer) was used for kidney SUVmax analysis.

### 3.1 Dose-rate and effective dose (ED) comparison

Ambient dose-rates were measured at the patient surface and at 1 m, immediately post-injection and at 1 h for each tracer. For [^68 Ga]Ga-PSMA-11, surface dose-rate declined from 148.4 to 69.5 µSv/h over the first hour, with corresponding 1 m values of 16.1–6.5 µSv/h (Table 1). For ^18F-choline, surface dose-rate declined from 123.7 to 58.2 µSv/h, with 1 m values of 14.6–5.7 µSv/h over the same interval (Table 2).

**Table 1:** Dose-rate (µSv/h) for ^68Ga-PSMA measured at the patient surface and at 1 m, immediately post-injection and at 1 h.

Distance from patient	Time after injection	Dose rate (μSv/h)	Effective dose (μSv)
Surface	Immediately (0 h)	148.4	148.4
Surface	1 h	69.5	69.5
1 m	Immediately (0 h)	16.1	16.1
1 m	1 h	6.5	6.5

Values reflect ambient dose-rate; ED_total (mSv) is analyzed separately.

Measured at surface level and 1 m, immediately after injection and 1-hour post-injection.

**Table 2:** Dose-rate (µSv/h) for ^18F-Choline measured at the patient surface and at 1 m, immediately post-injection and at 1 h.

Distance from patient	Time after injection	Dose rate (μSv/h)	effective dose (μSv)
Surface	Immediately (0 h)	123.7	123.7
Surface	1 h	58.2	58.2
1 m	Immediately (0 h)	14.6	14.6
1 m	1 h	5.7	5.7

Values reflect ambient dose-rate; ED_total (mSv) is analyzed separately.

Measured at surface level and 1 meter, immediately after injection and 1 hour post-injection.

For context on tracer biodistribution relevant to radiation-safety interpretation, kidney SUVmax distributions by tracer are summarized in Table 3 (pre-specified subset, *n* = 40 per tracer); these uptake metrics are used later for correlation analyses and should not be conflated with dose-rate or ED outcomes (Table 3).

**Table 3:** Kidney SUVmax by tracer: mean ± SD and range for ^68Ga-PSMA and ^18F-Choline.

Tracer group	Mean SUVmax (Kidneys)	Standard deviation (±SD)	Range (Min–Max)
^68Ga-PSMA-11	38.4	± 3.7	32.5–44.1
^18F-choline	9.6	± 1.2	8.0–11.1

VOIs were drawn on fused PET/CT over renal cortex using a standardized protocol; *n* = 40 per tracer.

**Figure 1:**
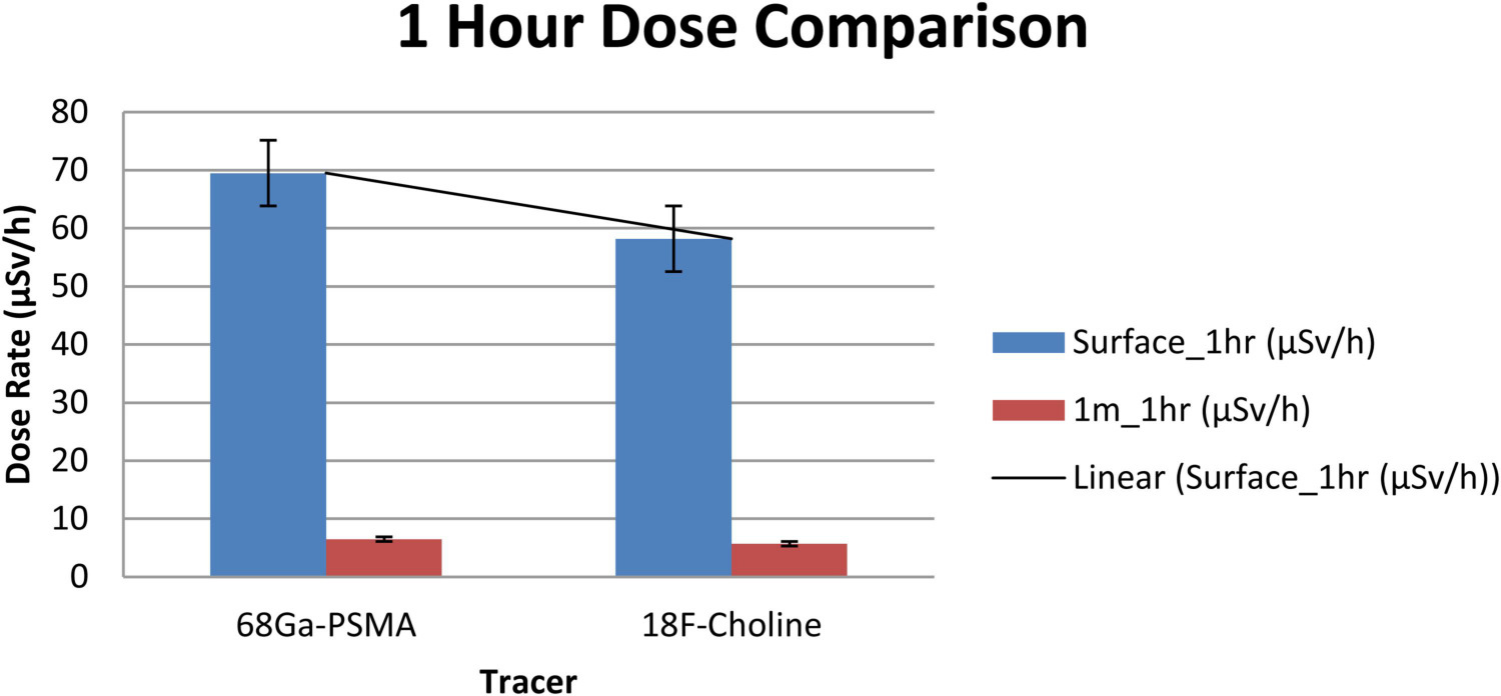
Ambient dose-rate (µSv/h) at 1 h post-injection for [^68 Ga]Ga-PSMA-11 vs. ^18F-choline at the surface and at 1 m (*n* = 70 per tracer). Bars show mean ± SD. Units: µSv/h.

Group comparisons at each geometry and timepoint are reported in Table 4. Immediately post-injection, dose-rates were significantly higher with [^68Ga]Ga-PSMA-11 than with ^18F-choline at both surface (148.4 vs. 123.7 µSv/h; *p* < 0.01) and 1 m (16.1 vs. 14.6 µSv/h; *p* < 0.05). At 1 h, surface dose-rates remained significantly different (69.5 vs. 58.2 µSv/h; *p* < 0.05), whereas the 1 m difference was not significant (6.5 vs. 5.7 µSv/h; *p* > 0.05) (Table 4). Figure 1 depicts the 1-h distributions at both distances for visual comparison.

**Table 4:** Statistical comparison of dose rates between tracers at each timepoint.

Measurement point	Tracer	Mean dose rate (μSv/h)	p-value (vs. another tracer)	Significance
Surface (0 h)	^68Ga-PSMA-11	148.4	**< 0.01**	Significant
	^18F-choline	123.7		
Surface (1 h)	^68Ga-PSMA-11	69.5	**< 0.05**	Significant
	^18F-choline	58.2		
1 m(0 h)	^68Ga-PSMA-11	16.1	**< 0.05**	Significant
	^18F-choline	14.6		
1 m (1 h)	^68Ga-PSMA-11	6.5	n.s. (*p* > 0.05)	Not Significant
	^18F-choline	5.7		

Bold *p*-values indicate statistically significant differences between tracers (*p* < 0.05).

Importantly, ambient dose-rate (µSv/h) is a point-in-time measurement and is not interchangeable with effective dose (ED, mSv) for the exam. In this cohort, ED_total (PET+CT) was similar between tracers (mean ± SD: ∼21.3 ± 3.6 mSv for [^68Ga]Ga-PSMA-11 vs. 20.7 ± 3.4 mSv for ^18F-choline; *p* = 0.28), consistent with tracer-specific dose coefficients and the uniform CT protocol used in this study. These ED results are analyzed separately to avoid endpoint mixing.

### 3.2 Correlation between body mass and dose rates

To check if radiation dose rates were linked to a patient's body mass, a separate correlation analysis was performed for every tracer. As shown in Figuress 2,3, scatter plots were created to show the relationship between body mass (kg) and dose rate (μSv/h) one hour after injection for ^68Ga-PSMA-11 and ^18F-choline. Patients with a higher body mass were observed to get slightly lower doses. The tracer's results indicated that the trend was not significant, implying that the differences in body mass among patients in the cohort were not primarily responsible for the observed variations. The data show that tracer behavior in the dose rate largely depends on how the drug is absorbed and removed, and not on body size.

**Figure 2:**
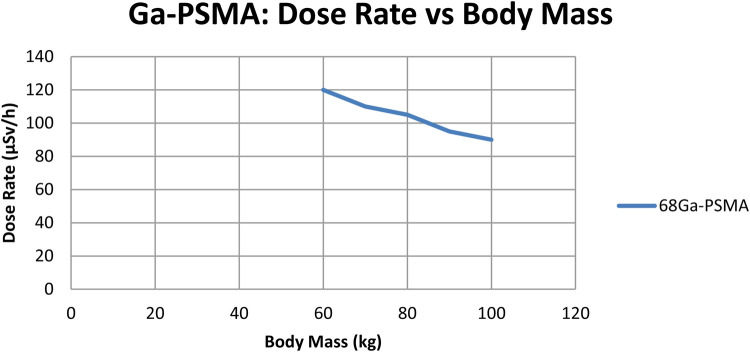
Dose-rate (µSv/h) at 1 h vs. body mass (kg) for ^68Ga-PSMA (*n* = 70); linear fit with 95% CI shown. Units: µSv/h, kg.

**Figure 3:**
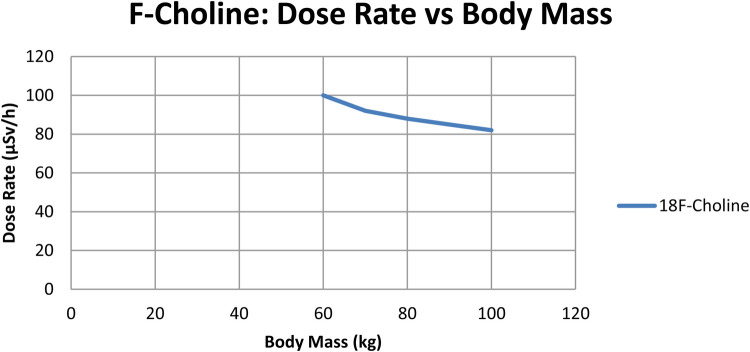
Dose-rate (µSv/h) at 1 h vs. body mass (kg) for ^68Ga-PSMA (*n* = 70); linear fit with 95% CI shown. Units: µSv/h, kg.

### 3.3 Suvmax and effective dose (ED) correlation (kidneys)

**Figure 4:**
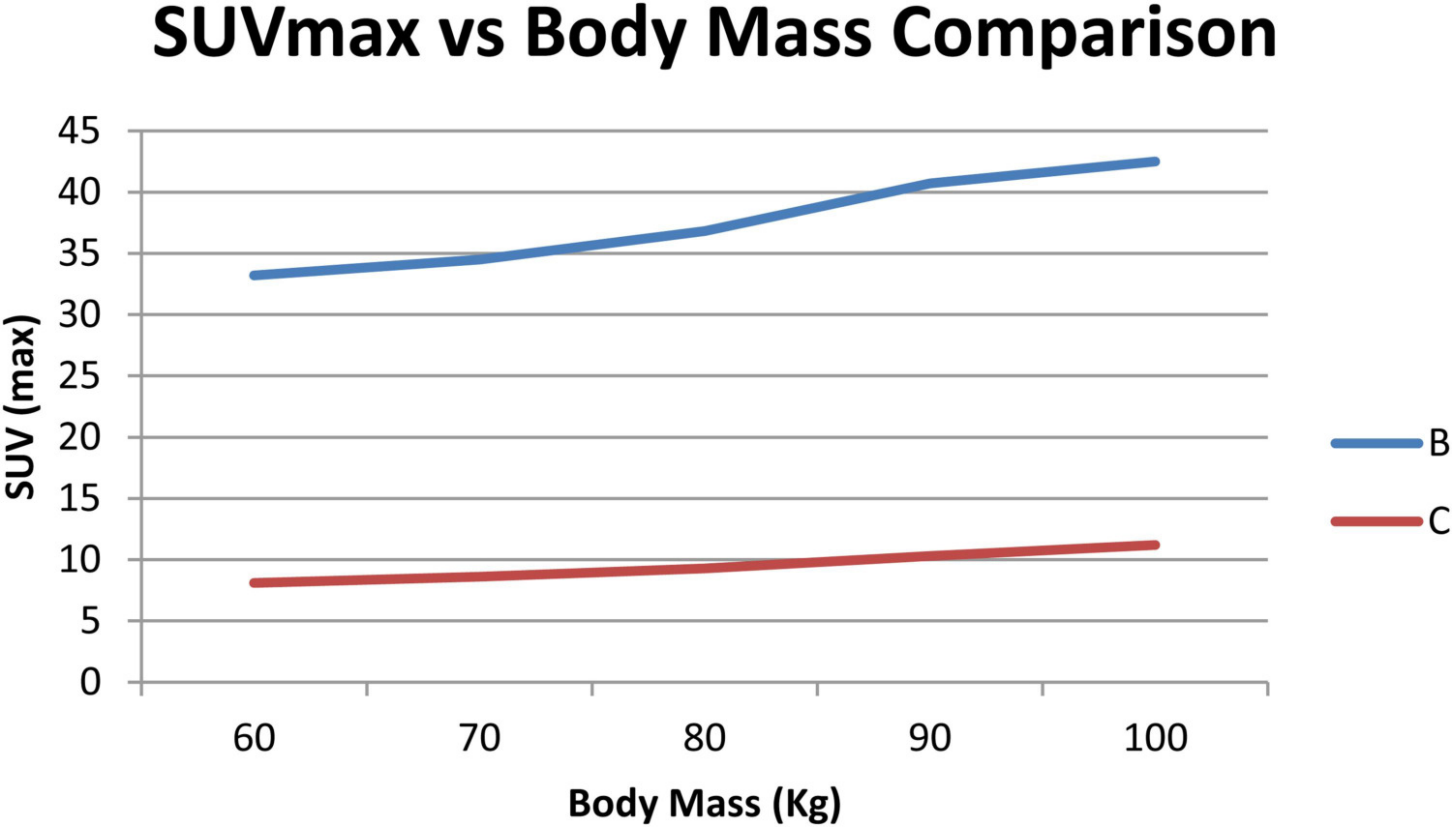
Summary chart showing SUVmax vs. dose trends between tracers.

We evaluated the prespecified association between kidney SUVmax and the exam effective dose ED *total* (ED *total* = ED_PET + ED_CT, in mSv). Baseline kidney SUVmax distributions by tracer are summarized in Table 3. For ^68Ga-PSMA, SUVmax correlated positively with ED *total* (*r* = 0.71; *p* < 0.001). For ^18F-choline, no significant association was observed (*r* = −0.12; *p* = 0.46). Analyses that involve ambient dose-rate (µSv/h) at fixed geometry/time are conceptually distinct from ED and are reported separately in the Supplementary Material (Table 5; Figure 4) to avoid endpoint mixing. During a consistency audit, the previously reported near-perfect negative coefficients (≈ −0.99) were traced to mismatched pairing of variables across timepoints/units; we re-computed correlations with correctly paired per-patient data.

**Table 5:** Correlation between kidney SUVmax and ED_total (mSv) for ^68Ga-PSMA and ^18F-Choline (Pearson's *r*, *p*-value; *n* = 40 per tracer). ED_total = ED_PET + ED_CT; ED metrics are distinct from dose-rate (µSv/h).

Tracer	*r*	*p*-value	Interpretation
^68Ga-PSMA	0.71	< 0.001	Moderate positive correlation
^18F-choline	−0.12	0.46	Not significant

## 4 Discussion

This study compared radiation-related metrics for prostate cancer PET/CT using [^68 Ga]Ga-PSMA and [^18F]F-Choline and examined whether kidney SUVmax is associated with the exam effective dose (ED_total, mSv), while characterizing ambient dose-rate (µSv/h) at standardized distance/time as an exploratory safety metric. Because ED_total (a whole-exam quantity driven by PET administered activity and CT protocol) and ambient dose-rate (a point-in-time measurement) quantify different constructs, their analyses were deliberately separated to avoid endpoint mixing.

Immediately post-injection, patients imaged with [^68Ga]Ga-PSMA exhibited higher ambient dose-rates than those imaged with [^18F]F-Choline (148.4 vs. 123.7 µSv/h). At 1 m, the corresponding values remained slightly higher for [^68Ga]Ga-PSMA (16.1 vs. 14.6 µSv/h). These differences are consistent with tracer-specific biodistribution and clearance patterns, PSMA ligands showing prominent renal handling and distinct target engagement, aligning with observations that exposure profiles can differ across tracers (Salah et al., 2021). From a practical standpoint, these early post-injection findings support reinforcing short-term distancing and workflow measures; however, dose-rate should not be interpreted as a surrogate for ED_total.

For the prespecified primary endpoint, kidney SUVmax demonstrated a moderate positive association with ED_total for [^68Ga]Ga-PSMA (*r* = 0.71; *p* < 0.001) and no significant association for [^18F]F-Choline (*r* = −0.12; *p* = 0.46). These results indicate that higher renal uptake on [^68Ga]Ga-PSMA studies may coincide with higher overall exam effective dose in our cohort, whereas such a relationship was not observed for [^18F]F-Choline. While prior work has described links between uptake metrics and absorbed dose in specific contexts (Khansa et al., 2020) and explored metabolic correlates of tracer handling (Bie et al., 2023) with tracer-dependent visibility and organ activity patterns (Beheshti et al., 2022), our data do not support using SUVmax as a stand-alone indicator of patient dose or safety. To maintain conceptual clarity, exploratory correlations involving ambient dose-rate are reported in the Supplementary Material, and should not be cross-interpreted with ED_total.

Operationally, the higher early dose-rates observed with [^68Ga]Ga-PSMA argue for emphasizing short-term time-and-distance precautions immediately after injection, within the framework of locally appropriate diagnostic reference levels and workflow policies (Said, 2025). In routine practice, ED_total is primarily governed by administered activity and CT acquisition parameters, with additional variability from patient habitus and uptake time; SUVmax may be considered contextual information about tracer behavior rather than a proxy for dose or a safety “check.” Methodological harmonization remains important for fair tracer comparisons (Huang et al., 2023).

This single-center design with a uniform scanner and timeline is a strength, as is the predefinition of the primary correlation endpoint and consistent units/definitions across analyses. Limitations include modest sample sizes for both the dosimetry and SUV subsets, the sensitivity of SUV to VOI definition, uptake time, and partial-volume effects, and the absence of lesion-level absorbed-dose mapping or pharmacokinetic modeling; residual confounding by administered activity, CT protocol, renal function, and body habitus cannot be excluded. Although SUV-based markers have been investigated for biological characterization and outcome associations in other settings (Fragkiadaki et al., 2024), the present findings should be regarded as hypothesis-generating rather than predictive.

Future work should include multi-center cohorts with harmonized timing, recovery-coefficient corrections, voxel-level dosimetry, and multivariable adjustment to determine whether organ-level SUV metrics add independent explanatory value beyond standard determinants of ED_total, and to refine tracer-specific operational guidance that integrates both ED and dose-rate data; advances in long axial field-of-view PET/CT may further enable robust kinetic and dosimetric assessments (Mingels et al., 2023). In summary, [^68 Ga]Ga-PSMA shows higher early ambient dose-rates than [^18F]F-Choline, and kidney SUVmax exhibits a moderate association with ED_total only for [^68Ga]Ga-PSMA in this cohort. These results do not support SUVmax as a stand-alone surrogate for radiation safety or as a predictor of therapeutic outcomes, reinforcing the need for clear separation of endpoints and cautious interpretation.

## 5 Conclusion

Across cohorts, early ambient dose-rate profiles differed modestly between tracers, higher immediately post-injection for ^68Ga-PSMA, whereas the average ED_total was similar. The observed associations between kidney SUVmax and dose metrics were limited and do not support using SUVmax as a stand-alone surrogate for radiation safety or clinical decision-making. These findings should be regarded as hypothesis-generating; confirmation in larger, harmonized, multi-center studies with standardized acquisition/reconstruction and voxel-level dosimetry is warranted. Operationally, routine time-and-distance precautions remain appropriate, with short-term reinforcement immediately after injection for ^68Ga-PSMA as a pragmatic measure pending further evidence.

## Data Availability

The original contributions presented in the study are included in the article/Supplementary Material, further inquiries can be directed to the corresponding author.

## References

[1] Abdi N Alsulami M Ghaznavi H. Comparing the diagnostic performance of [18F]PSMA-1007 with [68Ga]ga-PSMA-11 in PET/CT imaging and staging of recurrent prostate cancer. Med Adv. (2025) 3(1):9–19. 10.1002/med4.70006

[2] Agrawal A Natarajan A Mithun S Bakshi G Joshi A Murthy V Bone metastases in prostate cancer – gallium-68–labeled prostate-specific membrane antigen or fluorine 18 sodium fluoride PET/computed tomography – the better tracer? Nucl Med Commun. (2022) 43(12):1225–32. 10.1097/mnm.0000000000001621 36345767

[3] Alberts I Mingels C Zacho H Lanz S Schöder H Rominger A Comparing the clinical performance and cost efficacy of [68Ga]ga-PSMA-11 and [18F]PSMA-1007 in the diagnosis of recurrent prostate cancer: a Markov chain decision analysis. Eur J Nucl Med Mol Imaging. (2021) 49(12):4252–61. 10.1007/s00259-021-05620-9 34773473 PMC9525363

[4] Alonso Ó Santos G Fontes M Balter H Engler H. 68Ga-PSMA and 11C-choline comparison using a tri-modality PET/CT-MRI (3.0T) system with a dedicated shuttle. Eur J Hybrid Imaging. (2018) 2(1):1. 10.1186/s41824-018-0027-1 29782606 PMC5954786

[5] Beheshti M Taimen P Kemppainen J Jambor I Müller A Loidl W Value of 68Ga-labeled bombesin antagonist (RM2) in the detection of primary prostate cancer comparing with [18F]fluoromethylcholine PET-CT and multiparametric MRI—a phase I/II study. Eur Radiol. (2022) 33(1):472–82. 10.1007/s00330-022-08982-2 35864350 PMC9755087

[6] Bie K Veerman H Bodar Y Meijer D Leeuwen P Poel H Higher preoperative maximum standardised uptake values (SUVmax) are associated with higher biochemical recurrence rates after robot-assisted radical prostatectomy for [68Ga]ga-PSMA-11 and [18F]DCFPyL positron emission tomography/computed tomography. Diagnostics. (2023) 13(14):2343. 10.3390/diagnostics13142343 37510087 PMC10378114

[7] Bodar Y Veerman H Meijer D Bie K Leeuwen P Donswijk M Standardised uptake values as determined on prostate-specific membrane antigen positron emission tomography/computed tomography is associated with oncological outcomes in patients with prostate cancer. Br J Urol. (2022) 129(6):768–76. 10.1111/bju.15710 PMC9315142 35166426

[8] Fragkiadaki V Panagiotidis E Vlontzou E Kalathas T Paschali A Kypraios C Correlation of PSA blood levels with standard uptake value maximum (SUVmax) and total metabolic tumor volume (TMTV) in 18F-PSMA-1007 and 18F-choline PET/CT in patients with biochemically recurrent prostate cancer. Nucl Med Commun. (2024) 45(11):924–30. 10.1097/mnm.0000000000001881 39082074

[9] Huang S Ong S McKenzie D Mirabelli A Chen D Chengodu T Comparison of 18F-based PSMA radiotracers with [68Ga]ga-PSMA-11 in PET/CT imaging of prostate cancer—a systematic review and meta-analysis. Prostate Cancer Prostatic Dis. (2023) 27(4):654–64. 10.1038/s41391-023-00755-2 38017295 PMC11543591

[10] Khansa Z Neaimeh N Korek M Haidar M. Can SUVmax of 68Ga-labeled PSMA ligand and 18F-choline PET/CT be used to predict the radiation dose in prostate cancer patients? Health Phys. (2020) 120(1):80–5. 10.1097/hp.0000000000001287 32826522

[11] Laudicella R Torre F Davì V Crocè L Aricò D Leonardi G Prostate cancer biochemical recurrence resulted negative on [68Ga]ga-PSMA-11 but positive on [18F]fluoromethylcholine PET/CT. Tomography. (2022) 8(5):2471–4. 10.3390/tomography8050205 36287804 PMC9609559

[12] Lin C Lee M Lin C Kao C. Comparing the staging/restaging performance of 68Ga-labeled prostate-specific membrane antigen and 18F-choline PET/CT in prostate cancer. Clin Nucl Med. (2019) 44(5):365–76. 10.1097/rlu.0000000000002526 30888999

[13] Loureiro G Couto P González J Martínez Ó. Comparative evaluation of (18F)ALF-PSMA-HBED-CC and 68Ga-PSMA-HBED-CC in staging intermediate-/high-risk prostate cancer: a prospective study. World J Nucl Med. (2025) 24(2):118–27. 10.1055/s-0045-1801842 40336848 PMC12055253

[14] Mingels C Weidner S Sari H Buesser D Zeimpekis K Shi K Impact of the new ultra-high sensitivity mode in a long axial field-of-view PET/CT. Ann Nucl Med. (2023) 37(5):310–5. 10.1007/s12149-023-01827-y 36913094 PMC10129991

[15] Mutsuddy P Mandal T Akhter P Rahman S Siddique M Begum F 18F PSMA-1007 and 18F FDG PET/CT in an advanced prostate cancer patient – a case report. Bangladesh J Nucl Med. (2025) 28(1):174–7. 10.3329/bjnm.v28i1.79558

[16] Said A. Establishing local diagnostic reference levels for adult whole-body PET/CT in Malaysia. Radiat Prot Dosim. (2025) 201(8):568–76. 10.1093/rpd/ncaf053 40382712

[17] Salah H Almohammed H Mayhoub F Sulieman A Alkhorayef M Abolaban F Assessment of patient’s radiation exposures resulted from PET/CT 18F-FCH and 68Ga-PSMA procedures. Radiat Prot Dosim. (2021) 195(3–4):349–54. 10.1093/rpd/ncab077 34144608

[18] Seifert R Schafigh D Bögemann M Weckesser M Rahbar K. Detection of local relapse of prostate cancer with 18F-PSMA-1007. Clin Nucl Med. (2019) 44(6):e394–5. 10.1097/rlu.0000000000002543 30889005

[19] Shi C Yu K Hu Y Wang Y Bu F Ji L Diagnostic efficacy of various imaging modalities across different stages of prostate cancer: a network meta-analysis of diagnostic studies. medRxiv. (2024). 10.1101/2024.09.28.24314285

[20] Shi Y Wu J Xu L Zhu Y Wang Y Huang G The heterogeneous metabolic patterns of ganglia in 68Ga-PSMA, 11C-choline, and 18F-FDG PET/CT in prostate cancer patients. Front Oncol. (2021) 11:666308. 10.3389/fonc.2021.666308 33968772 PMC8103210

[21] International Commission on Radiological Protection. The 2007 recommendations of the international commission on radiological protection (ICRP publication 103). Ann ICRP. (2007) 37(2–4):1–332. 10.1016/j.icrp.2007.10.003 18082557

[22] International Commission on Radiological Protection. Radiation dose to patients from radiopharmaceuticals: a compendium of current information related to frequently used substances (ICRP publication 128). Ann ICRP. (2015) 44(2 Suppl):7–321. 10.1177/0146645314558019 26069086

[23] Deak PD Smal Y Kalender WA. Multisection CT protocols: sex- and age-specific conversion factors used to determine effective dose from dose-length product. Radiology. (2010) 257(1):158–66. 10.1148/radiol.10100047 20851940

[24] University of California, San Francisco Radiopharmaceutical Facility. Gallium Ga 68 PSMA-11 injection [Prescribing information; NDA 212643/212642]. U.S. Food and Drug Administration (2020). Available online at: https://www.accessdata.fda.gov/drugsatfda_docs/label/2020/212643s000lbl.pdf (Accessed March 16, 2021).

[25] Bolch WE Eckerman KF Sgouros G Thomas SR. Mird pamphlet No. 21: a generalized schema for radiopharmaceutical dosimetry—standardization of nomenclature. J Nucl Med. (2009) 50(3):477–84. 10.2967/jnumed.108.056036 19258258

[26] Stabin MG Sparks RB Crowe E. Olinda/EXM: the second-generation personal computer software for internal dose assessment in nuclear medicine. J Nucl Med. (2005) 46(6):1023–27. 10.2967/jnumed.104.034351 15937315

[27] Ceci F Oprea-Lager DE Emmett L Adam JA Bomanji J Czernin J E-PSMA: the EANM standardized reporting guidelines v1.0 for PSMA-PET. Eur J Nucl Med Mol Imaging. (2021) 48(5):1626–38. 10.1007/s00259-021-05245-y 33604691 PMC8113168

[28] Fendler WP Eiber M Beheshti M Bomanji J Calais J Ceci F Psma PET/CT: joint EANM procedure guideline/SNMMI procedure standard for prostate cancer imaging 2.0. Eur J Nucl Med Mol Imaging. (2023) 50(5):1466–86. 10.1007/s00259-022-06089-w 36604326 PMC10027805

[29] Soret M Bacharach SL Buvat I. Partial-volume effect in PET tumor imaging. J Nucl Med. (2007) 48(6):932–45. 10.2967/jnumed.106.035774 17504879

