# Correction: Comparative dosimetric evaluation of 68Ga-PSMA and 18F-Choline PET/CT imaging in prostate cancer: implications for radiation safety and SUVmax correlation

**DOI:** 10.3389/fnume.2025.1760153

**Published:** 2025-12-12

**Authors:** Hussein R. Kaafarani, Mohamad Haidar, Hanna El-Balaa

**Affiliations:** 1 Department of Nuclear Medicine, American University of Beirut Medical Center, Beirut, Lebanon; 2 Department of Nuclear Medicine, American University of Beirut (AUB), Beirut, Lebanon; 3 Medical Physics (Beirut/Hadath), Lebanese University, Beirut, Lebanon

**Keywords:** PET/CT, dosimetry, ^68Ga-PSMA, ^18F-Choline, prostate cancer, radiation safety, SUVmax

Author Hussein R. Kaafarani was erroneously spelled as Hussein Kaafarani.

The original version of this article has been updated.

