# Supplementary material for: Comparative dosimetric evaluation of 68Ga-PSMA and 18F-Choline PET/CT imaging in prostate cancer: implications for radiation safety and SUVmax correlation
Source: Front Nucl Med. 2025 Dec 1;5:1702390. doi: 10.3389/fnume.2025.1702390 (PMC12741963; doi:10.3389/fnume.2025.1702390)
Supplement: Supplementary file 2 [file Supplementaryfile1.docx]

**Comparative dosimetric evaluation of 68Ga‑PSMA and 18F‑Choline PET/CT imaging in prostate cancer: implications for radiation safety and SUVmax correlation**

Running title: Dosimetry of 68Ga‑PSMA vs 18F‑Choline

Manuscript type: Original Article

**Authors and affiliations**

- **Hussein Kaafarani**¹ — PhD
  ¹ Department of Nuclear Medicine, American University of Beirut Medical Center, Beirut, Lebanon
  **Correspondence address:** Beirut, Hadath, Saint Terrese, Donia Building, 5th floor
- **Mohamad Haidar **²
  ² American University of Beirut (AUB), Beirut, Lebanon
  **Email:** mh209@aub.edu.lb
- **Hanna El-Balaa **³ — Doctor, Medical Physics
  ³ Lebanese University, Medical Physics (Beirut/Hadath), Lebanon
  **Email:** hanna_balaa@yahoo.fr

Funding: None.

Conflicts of interest: The author declares no competing interests.

Ethical approval: Approved by the Institutional Review Board of AUBMC (approval no. XXX/2023); informed consent waived.

Data availability: De‑identified data available from the corresponding author upon reasonable request.

Acknowledgements: The author thanks the PET/CT technologists at AUBMC for assistance with dosimetry measurements.

Supplementary

**Table S2 (Supplement)**

**Table S2. Exploratory correlations between kidney SUVmax and dose-rate (µSv/h) at 1 m, ~1 h post-injection.**

| **Tracer** | **r** | **p-value** | **Interpretation** |
| --- | --- | --- | --- |
| ^68Ga-PSMA | -0.990 | 0.001 | Strong negative correlation |
| ^18F-choline | -0.992 | 0.0007 | Strong negative correlation |

**S1**: *Scatter plot with regression: SUVmax (kidney) vs. Dose Rate for Ga-PSMA*

**S2**: *Scatter plot with regression: SUVmax (kidney) vs. Dose Rate for F-Choline*
